# DUSP11 and triphosphate RNA balance during virus infection

**DOI:** 10.1371/journal.ppat.1009145

**Published:** 2021-01-14

**Authors:** Joon H. Choi, Christopher S. Sullivan

**Affiliations:** Department of Molecular Biosciences, LaMontagne Center for Infectious Disease, The University of Texas at Austin, Austin, Texas, United States of America; Mount Sinai School of Medicine, UNITED STATES

## Triphosphate RNA balance

**Fig 1:**
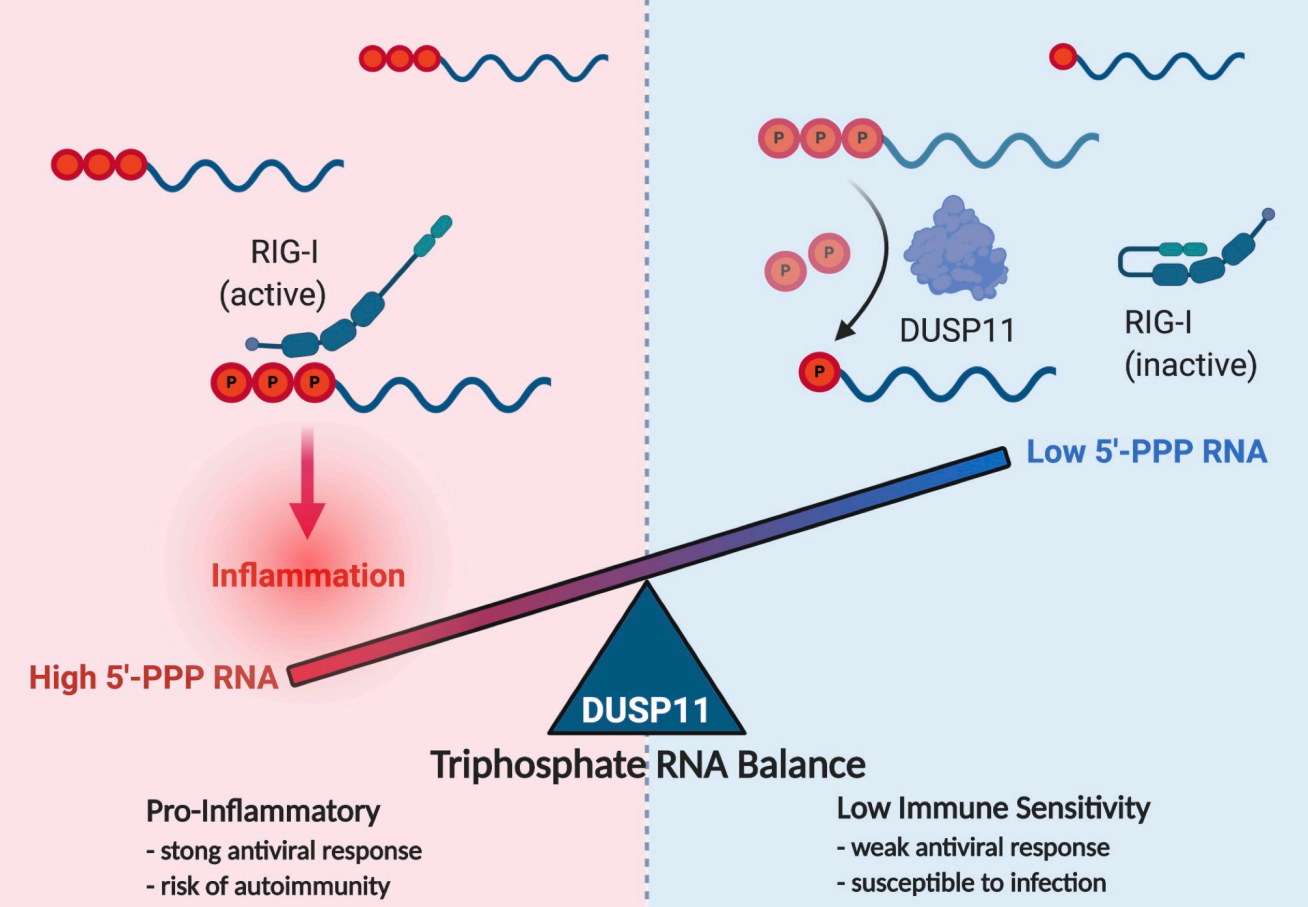
The concept of “triphosphate RNA balance”. Experiments with DUSP11 demonstrate that increasing or decreasing the cellular pool of 5′-PPP RNA modulates the sensitivity of the cell to activating the antiviral response. This argues that balancing the 5′-PPP RNA pool is essential for both preventing autoinflammation and initiating a timely and effective antiviral response. DUSP, dual-specificity phosphatase; 5′-PPP, 5′-triphosphate.

Most who study virus–host interactions are familiar with 5′-triphosphate (5′-PPP) RNA, a well-established pathogen associated molecular pattern (PAMP). 5′-PPP RNA activates the pattern recognition receptor (PRR) RIG-I leading to production of the antiviral type I interferons [1,2]. Less appreciated is the fact that essentially all host transcripts, of which there are millions per cell, initially are 5′-PPP RNA [3]. Some of these transcripts, particularly those transcribed by RNA polymerase III (RNAP III), are highly structured and remain 5′-PPP with the potential to activate RIG-I [4–6]. The mechanisms for how the cell prevents aberrant induction of an autoinflammatory response while remaining sufficiently sensitive enough to rapidly detect foreign RNA and trigger an effective antiviral response are now emerging. Studies reveal the importance of proper control of endogenous cellular triphosphate RNA levels, including appropriate subcellular localization and protein shielding of the 5′-end of host RNAP III transcripts to reduce exposure to RIG-I [7–10]. More recently, the cellular triphosphatase dual-specificity phosphatase (DUSP) 11 is emerging as an important player in maintaining triphosphate RNA balance [11–17]. Either increased or decreased levels of DUSP11 can decrease or increase, respectively, the propensity of a cell to undergo the antiviral response (Fig 1). The natural relevance of these observations and whether this mechanism can be exploited for therapeutic benefit are the subjects of ongoing interest.

## DUSP11 is a major regulator of cellular 5′-triphosphate RNA

DUSPs are a subfamily of protein tyrosine phosphatases (PTPs) that contain a highly conserved phosphatase catalytic domain (HCXXGXXR), which generally enables dephosphorylation of both phospho-serine/threonine and phospho-tyrosine [18]. DUSP11 is among the atypical DUSPs that contain the catalytic phosphatase motif but lacks the N-terminal domain known to confer specificity for protein substrates. Compared with other PTPs and DUSPs, purified DUSP11 displays poor (1 to 5 orders of magnitude less) activity toward generic protein substrates [19]. Unlike other DUSPs, DUSP11 possesses strong affinity for RNA in vitro [20]. Its catalytic phosphatase activity on generic 5′-PPP and 5′-PP RNA oligonucleotide substrates is high, approximately 2 to 3 orders of magnitude greater than on the most susceptible peptide substrate tested [19]. The atomic structure of DUSP11 reveals a larger active site crevice than that of protein phosphatases [21]. These features suggest that DUSP11 shares more in common with other RNA phosphatases such as the baculovirus phosphatase (BVP) and the phosphatase domain of the metazoan mRNA capping enzyme. Accordingly, cells lacking DUSP11 have an increase in the proportion of RNAP III-transcribed RNAs that are 5′-triphosphorylated [11]. These observations are consistent with the major biological activity of DUSP11 being an RNA triphosphatase.

## Changing the balance of host triphosphorylated RNA alters the propensity of cells to undergo an antiviral response

Transcriptome-wide analyses reveal that a subset of RNAP III transcripts (vault, RNA component of mitochondrial RNA processing (RMRP), Alu short interspersed nuclear element (SINE), and Y RNAs) are not only more likely to be triphosphorylated but are also more abundant in cells lacking DUSP11 [11]. Exposure of DUSP11-deficient cells to exogenous in vitro synthesized 5′-PPP RNA results in an enhanced increase in the expression of interferon-associated genes [15,17]. This phenotype requires RIG-I, a PRR that triggers the interferon response upon detection of structured 5′-PPP RNAs [17]. Consistent with these aberrant RNAs having biological impact, DUSP11-deficient mice display a signature of enhanced interferon signaling and show greater sensitivity to exogenous 5'-PPP RNA [17].

**Fig 2:**
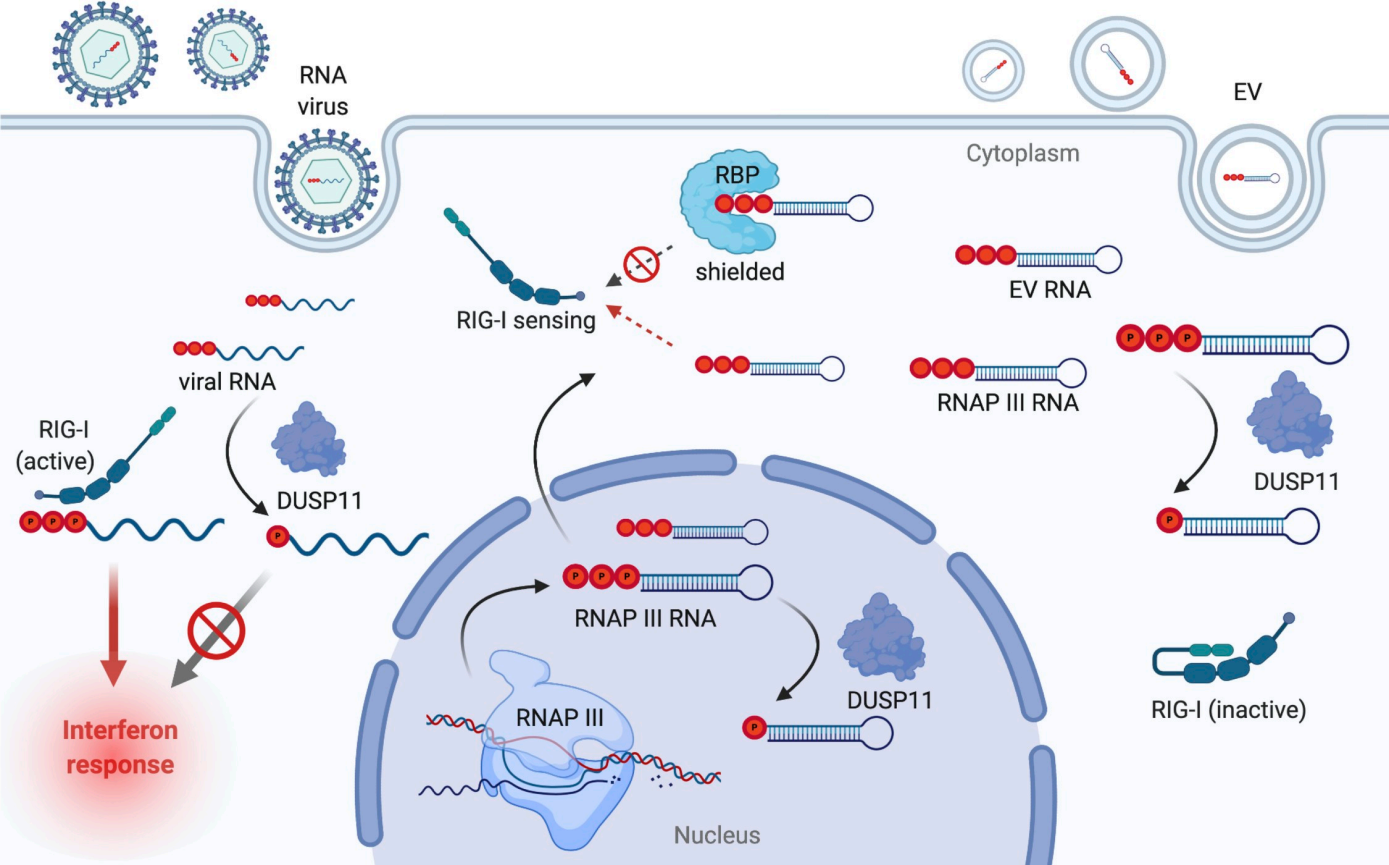
DUSP11 acts on host and viral 5′-triphosphate RNAs reducing their ability to stimulate an antiviral response. DUSP11 acts on RNAP III transcripts, possibly co-transcriptionally in the nucleus and upon delivery via extracellular vesicles (EV) in the cytosol, to prevent an inappropriate inflammatory response. Experimentally reducing DUSP11 leads to enhanced RIG-I signaling. Equally important is that increasing DUSP11 leads to attenuated RIG-I signaling and enhanced virus infection. RNA viruses may have evolved to allow their transcripts access to DUSP11 to reduce their visibility to host pathogen recognition receptors. DUSP, dual-specificity phosphatase; EV, extracellular vesicle; RNAP III, RNA polymerase III; RBP, RNA binding protein.

Induction of the cellular pool of 5′-PPP RNA accessible to RIG-I can be triggered by various biological stimuli and mechanisms and can increase the propensity of a cell to undergo an antiviral response. Herpes simplex virus-1 (HSV-1) infection depletes the levels of RNA binding proteins (RBPs) (thiosulfate sulfurtransferase (TST), mitochondrial ribosomal protein L18 (MRPL18)) that typically shield pseudogene 5S rRNA (RNA5SP141). This results in activation of RIG-I [7]. Infection with Kaposi’s sarcoma-associated herpesvirus (KSHV) or human immunodeficiency virus 1 (HIV-1) leads to reduced DUSP11 protein levels and increases in vault and Y RNA association with RIG-I [15,16]. Coculture of tumor cells with stromal cells can lead to increased RIG-I signaling via production of extracellular vesicles (EVs) enriched for 5′-PPP 7SL RNA cargoes. This depends on the altered stoichiometry of RBPs (signal recognition particle (SRP) 9 and SRP14) to 7SL RNA, which exposes unshielded 5′-PPP 7SL RNA to be packaged as EV cargo. The EV 7SL RNA cargo is subsequently detected by RIG-I in recipient tumor cells [8,9]. Interestingly, DUSP11 also participates in control of the inflammatory potential of EVs derived from tumor–stromal cell coculture [17]. Thus, triphosphate balance is maintained by multiple mechanisms including shielding of immunostimulatory RNAs by RBPs and dephosphorylating 5′-PPP RNAs by phosphatases such as DUSP11 (Fig 2). Altering this balance leads to the biological outcome of enhanced inflammation in disease states such as virus infection and cancer.

## DUSP11 can dephosphorylate viral transcripts to the benefit or detriment of the virus

5′-PPP RNAs are a molecular hallmark of RNA virus replication that are produced during the course of infection via viral RNA-dependent RNA polymerases (RdRPs) [6]. Cells and mice with reduced DUSP11 give rise to lower virus loads when infected with RNA viruses known to generate RIG-I agonist 5′-PPP-RNAs [17]. Although this phenotype is consistent with host RNAs priming RIG-I as described above, our work shows that DUSP11 can act directly on 5′-PPP viral-like RNAs known to activate RIG-I [14,17]. Infection of cells lacking DUSP11 with vesicular stomatitis virus (VSV) results in a higher overall abundance and proportion of 5′-triphosphorylated VSV leader RNA, a well characterized small RNA RIG-I agonist [22–24]. These data suggest that DUSP11 can be usurped by some RNA viruses to reduce the visibility of viral transcripts to RIG-I (Fig 2).

Separately, some viral 5′-PPP RNAs have a role in mammalian RNA gene silencing. Select noncanonical viral precursor microRNAs (pre-miRNAs) transcribed by RNAP III utilize DUSP11 phosphatase activity to promote RNA silencing activity [11,25]. DUSP11 converts the 5′-PPP microRNA (miRNA) precursors to a 5′-P, which is required for efficient loading of derivative miRNAs into the miRNA RNA induced silencing complex (miRISC). While it remains unknown which, if any, host small RNAs depend on DUSP11 for silencing activity, promoting viral miRNA activity is an additional example in which a host RNA phosphatase can benefit the life cycle of some viruses.

Although DUSP11 is beneficial to some viruses, DUSP11 activity is not always pro-viral. For viruses such as the hepatitis C virus (HCV) that are sensitive to 5′-3′ exoribonuclease (XRN)-mediated restriction, the triphosphatase activity of DUSP11 can be antiviral. Mechanistically, DUSP11 converts the 5′-PPP HCV RNA to 5′-P and renders it susceptible to the monophosphate-specific XRN exonuclease [13,14]. In addition to DUSP11, a different triphosphatase, DOM3Z (DXO), can also act on HCV transcripts rendering them susceptible to XRN attack [13]. This brings up an important point that triphosphatases other than DUSP11 may also take part in maintaining cellular triphosphate RNA balance in other contexts. Defining these factors, and in which contexts they are pro- or antiviral, should illuminate new ways forward for altering triphosphate RNA balance for therapeutic good.

## Major unresolved questions in the nascent field of triphosphate RNA balance

Although multiple groups have contributed to the understanding of triphosphate RNA balance and the role of DUSP11 [11,13–17] key questions remain unanswered. Perhaps foremost is whether DUSP11 activity levels are naturally controlled to tune the propensity of a cell to undergo antiviral signaling. At least part of the mechanism by which HIV infection reduces DUSP11 levels resulting in enhanced RIG-I signaling appears to be through the activity of the viral-encoded viral protein R (VPR) protein. This implies that enhanced RIG-I signaling is somehow advantageous to HIV [16]. However, it remains possible that the host response to infection can contribute to the negative control of DUSP11 that occurs during infection with HIV and other viruses. Consistent with this, activation of KSHV lytic infection from a latent state reduces cellular DUSP11 levels, coincident with increased RIG-I signaling [15]. Further, VSV infection also results in altered subcellular localization and reduced overall steady-state levels of DUSP11 [17]. Thus, DUSP11 levels are reduced by infection with diverse virus families, implying advantage to the host by increasing the propensity of cells to activate the antiviral response.

The bulk of DUSP11 protein is nuclear. Yet, a small fraction of DUSP11 is cytosolic in all situations that have so far been examined [12,13,17]. This brings up another aspect of DUSP11 biology that remains unresolved. What is the natural function of cytosolic DUSP11? The fact that DUSP11 is known to directly act on cytoplasmic-restricted HCV and VSV viral transcripts demonstrates that cytosolic DUSP11 is catalytically active. This raises the interesting possibility that a natural function for cytoplasmic DUSP11 exists in uninfected cells. One idea is that EVs that contain RNAP III-derived transcripts [26,27] may be “sensed” by receiving cells to enact a DUSP11-dependent control mechanism. Consistent with this, EV 5′-PPP RNA cargo is affected by DUSP11 both in the EV-producing and EV-receiving cells [17]. Therefore, if EVs are situationally PAMPogenic depending on their 5′-PPP cargoes, then the DUSP11-mediated control could exist to reduce the possibility of toxic autoinflammatory responses. When pathogens are detected, cells may reduce DUSP11 activity to increase the propensity of neighboring cells to trigger an antiviral state. If so, this would represent a previously unappreciated cell-to-cell communication system that can tune the RIG-I response. Conversely, viruses that enter via endocytosis may have evolved to take advantage of this DUSP11 activity for their own benefit. These ideas warrant further investigation.

Another open question regarding DUSP11 is the possibility that it has substrates other than RNA. DUSP11 shares a common ancestor with a family of protein phosphatases [21]. Recently, it was reported that DUSP11 acts on the TGF-β activated kinase 1 (TAK1) protein preventing a lethal inflammatory response of mice that are exposed to the bacterial PAMP lipopolysaccharide (LPS) [28]. Further, Cai and colleagues show that DUSP11 can promote proliferation in cellular transformation assays, which they speculate could be due to protein substrates of DUSP11 [29]. However, given that the DUSP11 catalytic site structure is typical of RNA phosphatases, and that DUSP11 shows preferentially enhanced activity on RNA over peptide substrates [19–21], it is unclear how large a role peptide substrates will play in the biology associated with DUSP11. Although a biologically relevant protein substrate cannot be ruled out, it is interesting to note that the increased RIG-I signaling induced by reduced DUSP11 [17] would also be expected to enhance phospho-TAK1 [30,31]. Further, activated RIG-I is a known negative regulator of cell proliferation [32,33]. Therefore, it will be important to determine if RIG-I and/or 5′-PPP RNA is involved in the enhanced LPS-mediated inflammatory response or the reduced cellular transformation associated with ablation of DUSP11.

The chronic low-level interferon response of DUSP11-deficient mice presents an opportunity to explore how such a state alters the antiviral response and the plausibility of inducing or preventing such a state for therapeutic benefit. Such therapies could involve reducing DUSP11 or the activity of other RNA phosphatases for defense against pathogens and cancers. Alternatively, enhancing RNA phosphatase activities may ameliorate inflammatory disease. To pursue these ideas, it would be beneficial to define all the relevant players involved in triphosphate RNA balance and develop a deeper understanding of the mechanisms by which they function. The emerging understanding of triphosphate RNA balance and its relevance to the host response to infection promise a fruitful near future, replete with new insights into controlling infection and the innate immune response.
